# Nutritional Status of HIV-positive Patients in Niterói, Rio de Janeiro, Brazil

**Published:** 2014-12

**Authors:** Andrea F. Kroll de Senna, Solange A. de Oliveira, Luis G.C. Velarde, Sérgio Setúbal

**Affiliations:** ^1^Programa de Pós-Graduação em Ciências Médicas–Universidade Federal Fluminense–UFF; ^2^Serviço de Infectologia, Hospital Universitário Antônio Pedro–HUAP; ^3^Departamento de Estatística–Universidade Federal Fluminense–UFF

**Keywords:** Acquired immunodeficiency syndrome, Cross-sectional studies, HIV, Nutritional status, Obesity, Brazil

## Abstract

The objective of this cross-sectional study was to assess the nutritional status of HIV-positive patients in a hospital in the city of Niterói, Rio de Janeiro, Brazil. We studied 235 patients (130 men and 105 women) from May 2009 to June 2010. The frequency of undernourishment among women was 7.6%; 26.7% of the women were overweight, and 16.2% were obese. Among men, the frequency of undernourishment was 3.8%; 25.4% of the men were overweight, and 6.9% were obese. A logistic regression was done to investigate the relationship between nutritional status and potential predisposing factors. Women were more frequently affected by obesity and undernourishment than men. However, only the difference in obesity was significant, and women had almost three times higher odds of being obese (OR 2.6; 95% CI 1.03-6.65). According to a nationwide survey done in Brazil during 2008-2009, 50.1% of the Brazilian healthy males were overweight, and 12.5% were obese; 48% of healthy females were overweight, and 16.9% were obese. Although the prevalence of undernourishment in HIV-positive patients is now lower than that observed in the beginning of the AIDS epidemic and excess weight is increasingly common among people living with HIV/AIDS, the proportion of excess weight was found lower and of undernourishment was higher in the present study than that found in the Brazilian population.

## INTRODUCTION

More than 600,000 AIDS cases have been reported in Brazil since the appearance of the first patient in 1980 (1). Around 180,000 HIV-infected Brazilian patients are under combined antiretroviral therapy (cART), taking drugs supplied by the Ministry of Health and distributed allover the country through a network of public hospitals (2). As happened elsewhere, the incidence of opportunistic infections and neoplasms has declined since the widespread introduction of cART (3,4), and these are no longer the main causes of death in Brazilian AIDS patients, having been replaced by liver disease, a complication related to, or worsened by, antiretroviral drugs (5). Other problems probably due to these drugs are the increase in circulating lipids and fatty mass, obesity and fat redistribution, and metabolic disturbances that may lead to insulin resistance, diabetes mellitus, and, ultimately, to increased risk of cardiovascular disease (5-12).

Early in the epidemic, it was common for AIDS patients to present with a story of involuntary weight loss, and as many as 40-90% of them were importantly undernourished, a condition related to increased mortality (13-16). The nutritional status of these patients changed remarkably with the advent of cART in the 1990s (13), and a high prevalence of overweight and obesity is now observed (17-21).

We studied the nutritional status of HIV-positive outpatients in a public hospital in the city of Niterói, Rio de Janeiro State, Southeastern Brazil. We looked for differences in their nutritional status that might be related to sex and other variables and compared their nutritional status with those found in other studies carried out with HIV seropositive patients and in the Brazilian population at large.

## MATERIALS AND METHODS

This cross-sectional study was carried out from May 2009 to June 2010 and involved AIDS/HIV-positive outpatients of both sexes and aged over 20 years, who attended the Infectious Diseases Service of Antônio Pedro University Hospital (HUAP), the largest public hospital (250 beds) in the city of Niterói, Rio de Janeiro State, Southeastern Brazil. Patients were prospectively and consecutively enrolled during routine outpatient care. Pregnant women were excluded. All data were collected by one of the authors (AFKS). The study was approved by the HUAP Committee of Ethics in Research under the number CEP CMM/HUAP 041/09. All participants gave informed consent signing a form.

The main variables studied were sex, age, weight, height, and body mass index (BMI). Nutritional status was defined according to BMI (22). The patients were weighed and measured by a nutritionist. Individuals were weighed barefoot and with as little clothing as possible, usually in the morning. Stature was measured with a portable stadiometer attached to a wall. Subjects were positioned barefoot against the wall, with their feet together, arms hanging at their sides, and head, heels, and buttocks leaning against the wall. People were considered obese when their BMI was equal to or greater than 30 kg/m^2^, overweight when their BMI was between 25 and 29.9 kg/m^2^, normal when their BMI was between 18.5 and 24.9 kg/m^2^, and undernourished when their BMI was below 18.5 kg/m^2^. We also studied the possible relationships of the nutritional status, with several other variables, namely ethnic group, educational level, income, smoking habits, alcohol consumption, hypertension, physical activity, previous opportunistic diseases, viral load, CD4+ T cell counts, glucose, triglyceride and cholesterol levels. We also assessed the use of antiretroviral and other prescribed drugs, how long these were taken, and the duration of HIV infection. Family history of diabetes, hypertension, cardiovascular diseases, and dyslipidaemia was also recorded. Data were obtained with a questionnaire and from the medical records.

In statistical bivariate analysis, we used Pearson's chi-square and ANOVA tests for categorical and numeric variables respectively. A p value of <0.05 was considered significant. For ascertaining the influence of the variables on nutritional status, with p values of ≤0.1, we performed a logistic regression with Statistical Package for Social Science (SPSS) software (version 17.0). For odds ratios and sample-sizes, we used the Epi Info software (version 6.4).

## RESULTS

We studied 235 patients who presented from 14 May 2009 to 17 June 2010 for viral load measurements and CD4+ T cell counts. The mean age was 43.1 years, ranging from 20 to 75 years. Most patients were between 41 and 50 years of age. Men tended to be older than women as 69.2% of the men and 55.2% of the women were over 40 years. The mean CD4+ T cell count was 539 (ranging from 6 to 2,181). The HIV viral load was measured in 215 patients. It was undetectable in 153 patients and above 100,000 copies/mL in 14 patients.

As to the nutritional status, the percentages of malnutrition, normal weight, overweight, and obesity were 5.5%, 57.4%, 26.0%, and 11.1% respectively (Table 1). Overall, 37.1% of the study patients had excess weight, either overweight or obesity. Women were more frequently obese (16.2%) and underweight (7.6%). However, only the difference in obesity was significant, and women had almost three times higher odds of being obese (OR 2.6; 95% CI 1.0-36.65). There were no significant differences in the mean BMI between the sexes (ANOVA, F=1.813, p=0.179).

In bivariate analysis, only age <40 years, female sex, low educational level, and hypertension had a significant relationship with excess weight. This was not found with any other variable studied. A regression analysis was carried out to investigate the possible interrelationships between nutritional status and the variables, with p values of <0.1 in the bivariate analysis. In this multivariate analysis, age <40 years, hypertension, and smoking were still relevant. Female sex and hypertension raised the odds of having excess weight by a factor of 6.76 and 13.12 respectively (Table 2).

## DISCUSSION

Obesity appears to be more common among women, no matter if they are HIV-positive or not. Indeed, Amorosa *et al*. (17), studying American seropositive patients, observed that the frequency of overweight was almost the same among men and women but women had an almost three times higher chance of being obese, and the prevalence of excess weight (either overweight or obesity) was 58.2% among women and 42.3% among men. Likewise, a US study (18), also studying American seropositive patients, described gender differences in excess weight present in 52.1% of men and in 62.5% of women. In a study carried out in Porto Alegre, a city in the most Southernmost region of Brazil, Kroll *et al*. (20) observed, among seropositive patients, rates of obesity of 4.4% in men and of 14.1% in women (OR 3.53; 95% CI 1.47-8.69). According to the Family Budget Survey (FBS)—a nationwide survey done in Brazil in 2008-2009 (23)—50.1% of the Brazilian healthy males were overweight, and 12.5% were obese; 48% of healthy females were overweight, and 16.9% were obese. Thus, Brazilian men had a slightly larger percentage of overweight but women had a considerable higher frequency of obesity. In Southeastern Brazil, where the city of Niterói is located, percentages of overweight and obesity in the healthy urban population followed the Brazilian profile and were 52.4% and 13% among men, and 48.5% and 17.5% among women.

**Table 1.** Distribution of the HIV-seropositive patients according to the nutritional status and sex, Niterói, Rio de Janeiro, Brazil, 2009-2010

Parameter	Sex					
	Male		Female		Total	
Nutritional status	N	*%*	N	*%*	N	*%*
Underweight	5	3.8	8	7.6	13	5.5
Normal	83	63.8	52	49.5	135	57.4
Overweight	33	25.4	28	26.7	61	26
Obesity	9	6.9	17	16.2	26	11.1
Total	130	100	105	100	235	100

χ^2^=8,114; p=0.044

**Table 2.** Variables associated with excess weight among HIV-seropositive patients in Niterói, Rio de Janeiro, Brazil, 2009-2010

Parameter	OR	95% CI	p value
Hypertension	13.118	1.759-97.807	0.012
Female sex	6.758	1.786-25.581	0.005
Age <40 years	0.203	0.053-0.775	0.020
Smoking	0.245	0.061-0.986	0.048

Like obesity, undernourishment appeared to be more common among women, no matter if they were HIV-positive or not. The prevalence of underweight in seropositive patients seems now to be much lower than in the beginning of the AIDS epidemic when 40-90% of patients had important malnutrition (13-16). Nevertheless, Amorosa *et al*. (17), who were the first to show that obesity and overweight were more prevalent than malnourishment among seropositive patients, found a still considerably high percentage of undernourished patients (9%). According to the FBS (23), the percentages of malnutrition in the Brazilian healthy population were 1.8% in men and 3.6% in women. In cities of the Southeastern region of Brazil, percentages of malnutrition were 1.4% in men and 3.1% in women (23). In Niterói, Bossan *et al*. (24) found that the percentages of malnutrition in the healthy population were 2.2% in men and 4.4 in women.

The mean BMI found in the present study was also higher among women (24.2 kg/m^2^ vs 23.9 kg/m^2^), who were more frequently obese than men (16.2% vs 6.9%; OR 2.6; 95% CI 1.03-6.91). Female patients also had higher prevalence of malnutrition than men (7.6% vs 3.8%). The odds of women being malnourished were also higher but not statistically significant (OR 2.06; 95% CI 0.64-7.10).

Interestingly, the percentages of excess weight found in the present study (32.3% in men and 42.9% in women) were lower than those described by Bossan *et al*. (24) in the healthy population of Niterói (49.6% in men and 45.8% in women), lower than those in the Brazilian healthy population (between 62.6% and 64.9%), and lower than those in the healthy population of the Southeastern region (between 65.4% and 66%) as described in the FBS (23).

On the contrary, the percentages of undernourishment in our HIV-positive patients (3.8% in men and 7.6 in women) were higher compared to those found among other HIV-positive patients (17-21) and in the Brazilian healthy population (23,24).

Kroll *et al*. (20) also observed, among seropositive patients studied in Porto Alegre, higher percentages of underweight and lower percentages of overweight and obesity than those found in the healthy population of Brazil's Southern region. Similar results were found in all studies of Brazilian HIV-positive patients reviewed by us, in which rates of underweight were higher and rates of excess weight lower than those in the healthy population (17-21,23-26).

Some factors were associated with obesity. In this study, excess weight related significantly to women aged below 40 years, low educational level, hypertension and, in a multivariate analysis, to smoking. Being women and having high blood pressure or low educational level raised the odds of having excess weight but being a smoker and over 40 years of age reduced those odds. Smoking had a protecting effect against weight gain (27). Excess weight being less frequent in patients over 40 years is probably related to the progression of AIDS. We could not find any factor that was clearly associated with underweight, a probable consequence of morbid conditions.

### Limitations

There are some limitations in our study. The prevalence of underweight found (5.5%) is consistent with the expected proportion (5%) of underweight individuals in any population (24). This is not the proportion in the Brazilian population, however. Although our sample-size was sufficient for detecting prevalence differences between our patients and people in Brazil as a whole, or between our patients and individuals in Southeastern Brazil, it was insufficient for detecting differences between undernourished people among our patients and those in the population of Niterói. In spite of some concerns about the accuracy of BMI in detecting body adiposity (28), this index is still widely used and permits straightforward comparisons with other studies (29).

### Conclusions

Against the common belief that HIV-positive patients are now more overweight than the general population, the frequencies of overweight and obesity in this study were lower, and the frequency of undernourishment was higher than in the population at large. Malnutrition and obesity were more common among women. Factors associated with excess weight were the feminine sex, age below 40 years, low educational level, hypertension, and being a non-smoker.

## ACKNOWLEDGEMENTS

This work was possible through the support of Hospital Universitário Antônio Pedro; Programa de Pós-graduação em Ciências Médica da Universidade Federal Fluminense; and Coordenação de Aperfeiçoamento de Pessoal de Nível Superior (Capes), from which A.F. Kroll de Senna received a scholarship grant. We are also grateful to the patients who took part in this investigation.
